# Similar Adverse Pregnancy Outcome in Native and Nonnative Dutch Women with Pregestational Type 2 Diabetes: A Multicentre Retrospective Study

**DOI:** 10.1155/2013/361435

**Published:** 2013-10-30

**Authors:** Bart Groen, Thera P. Links, Paul P. van den Berg, Marieke Hellinga, Sharon Moerman, Gerard H. A. Visser, Wim J. Sluiter, Marijke M. Faas, Manon C. J. Schreuder, Willy Visser, Petronella H. L. M. Geelhoed-Duijvestijn, Rutgert Bianchi, Anton K. M. Bartelink, Harold W. de Valk

**Affiliations:** ^1^Department of Endocrinology, University Medical Centre Groningen, University of Groningen, Hanzeplein 1, 9713 GZ Groningen, The Netherlands; ^2^Department of Obstetrics and Gynaecology, University Medical Centre Groningen, University of Groningen, Hanzeplein 1, 9713 GZ Groningen, The Netherlands; ^3^Department of Endocrinology, University Medical Centre Utrecht, Heidelberglaan 100, 3584 CX Utrecht, The Netherlands; ^4^Department of Obstetrics and Gynaecology, University Medical Centre Utrecht, Heidelberglaan 100, 3584 CX Utrecht, The Netherlands; ^5^Division of Medical Biology, University Medical Centre Groningen, University of Groningen, Hanzeplein 1, 9713 GZ Groningen, The Netherlands; ^6^Department of Internal Medicine, Academic Medical Centre, Meibergdreef 9, 1105 AZ Amsterdam, The Netherlands; ^7^Department of Internal Medicine, Erasmus Medical Centre, 's-Gravendijkwal 230, 3015 CE Rotterdam, The Netherlands; ^8^Department of Internal Medicine, Medical Centre Haaglanden, Lijnbaan 32, 2512 VA The Hague, The Netherlands; ^9^Department of Endocrinology, Atrium Medical Centre, Henri Dunantstraat 5, 6419 PC Heerlen, The Netherlands; ^10^Department of Endocrinology, Meander Medical Centre, Ringweg Randenbroek 110, 3816 CP Amersfoort, The Netherlands

## Abstract

*Objective*. To assess the incidence of adverse pregnancy outcome in native and nonnative Dutch women with pregestational type 2 diabetes (T2D) in a multicenter study in The Netherlands. *Methods*. Maternal characteristics and pregnancy outcome were retrospectively reviewed and the influence of ethnicity on outcome was evaluated using independent *t*-test, Mann-Whitney *U*-test, and chi-square test. *Results*. 272 pregnant women (80 native and 192 non-native Dutch) with pregestational T2D were included. Overall outcome was unfavourable, with a perinatal mortality of 4.8%, major congenital malformations of 6.3%, preeclampsia of 11%, preterm birth of 19%, birth weight >90th percentile of 32%, and a Caesarean section rate of 42%. In nonnative Dutch women, the glycemic control was slightly poorer and the gestational age at booking somewhat later as compared to native Dutch women. However, there were no differences in incidence of preeclampsia/HELLP, preterm birth, perinatal mortality, macrosomia, and congenital malformations between those two groups. *Conclusions*. A high incidence of adverse pregnancy outcomes was found in women with pregestational T2D, although the outcome was comparable between native and non-native Dutch women. This suggests that easy access to and adequate participation in the local health care systems contribute to these comparable outcomes, offsetting potential disadvantages in the non-native group.

## 1. Introduction

Pregestational diabetes mellitus comprises both type 1 and type 2 diabetes mellitus. Pregestational type 1 diabetes mellitus is clearly associated with an increased incidence of adverse maternal, fetal, and neonatal outcome [1–4], and several studies in the last two decades have shown that pregestational type 2 diabetes poses an emerging problem, with pregnancy outcomes at least as poor as in women with type 1 diabetes [5–9]. This gains even more importance in view of the global diabetes epidemic which leads to ever increasing numbers of women in the childbearing age with pregestational type 2 diabetes [5, 10]. To add to the problem, pregestational type 2 diabetes is encountered frequently in specific subpopulations in north-western Europe, such as recently migrated women from Africa, Asia, and the Middle East [11]. These women are possibly more prone to suboptimal participation in the health care system because of frequently existing language barriers, generally less financial resources, and low education levels in those immigrant groups.

The Netherlands can be considered as a representative developed European country with ethnic minorities reflecting economic and social history [12]. Data from an epidemiological study from the Netherlands revealed that perinatal mortality is increased in women with a nonnative Dutch origin [13]. Therefore, the question rises if ethnicity plays a role in the development of pregnancy complications in women with type 2 diabetes, since the incidence of type 2 diabetes is more common in specific ethnicities [11]. Unfortunately, the possible relation between ethnicity and pregnancy complications in women with type 2 diabetes has received limited attention in western Europe. Data from two British studies showed no differences in pregnancy outcome between native British and one single other ethnic (i.e., Afro-Caribbean or Indo-Asian) women with type 2 diabetes [14, 15]. However, these studies were not nationwide and compared no mixture of ethnicities. Therefore, to elucidate more comprehensively the potential impact of ethnic origin (and their possible accompanying problems) on pregnancy outcome, we performed a retrospective multicentre study to assess maternal, fetal, and neonatal outcome in a large group of native and a mixture of nonnative Dutch women with pregestational type 2 diabetes from different hospitals spread throughout the Netherlands. We hypothesized that nonnative Dutch women with type 2 diabetes had a more unfavourable pregnancy outcome as compared to native Dutch women with type 2 diabetes.

## 2. Methods

### 2.1. Patients

A multicentre, retrospective study was performed involving seven large hospitals (University Medical Centre Groningen, University Medical Centre Utrecht, Academic Medical Centre Amsterdam, Erasmus Medical Centre Rotterdam, Medical Centre Haaglanden the Hague, Atrium Medical Centre Heerlen, and Meander Medical Centre Amersfoort) spread around the Netherlands. All women with pregestational type 2 diabetes are referred to hospital care in the Netherlands. 272 women in whom a singleton pregnancy progressed beyond 20 weeks of gestation and who delivered between January 1997 and August 2009 were included in an anonymised database and subsequently evaluated. 

The diagnosis pregestational type 2 diabetes was accepted when patients were anti-GAD antibody negative and/or had never experienced a keto-acidotic episode and the diabetes was being managed with diet alone or oral blood glucose-lowering agents and/or insulin. In the latter case, the diagnosis of type 2 diabetes was accepted when treatment with insulin was initiated >6 months after the initial diagnosis.

Patients were divided into native and nonnative Dutch groups, which was representative for the socioeconomic history of the Netherlands. Patients in the latter group were of North-African, Hindu, Afro-Caribbean, Asian, or other nonnative origin.

### 2.2. Methods

We retrospectively reviewed all charts and recorded maternal characteristics (age, body mass index, ethnic origin, alcohol use, smoking habits, and parity), duration of diabetes, presence of chronic complications, and preconceptional treatment of diabetes. To assess the potential impact of ethnicity on pregnancy outcome, the origin of the pregnant women was classified as (1) native Dutch and (2) nonnative Dutch. The size of the different ethnic groups (i.e., North-African, Hindu or Afro-Caribbean, etc.) was too small for adequate statistical analysis of these specific ethnicities. HbA1c (mmol/mol) values were recorded and median HbA1c was calculated in the periods one year before pregnancy and during the first, second, and third trimesters. 

### 2.3. Outcome Measures

#### 2.3.1. Obstetric Complications

Preeclampsia was defined as a diastolic blood pressure ≥90 mmHg on two occasions at least four hours apart in the second half of pregnancy in previously normotensive women and de novo albuminuria (≥300 mg/24 h) [16]. In women with preexisting hypertension, pre-eclampsia was diagnosed when albuminuria de novo occurred in the second half of pregnancy. HELLP syndrome was defined as platelet count ≤100 · 10^9^/L, elevated liver enzymes (serum alanine aminotransferase >70 U/L and/or serum aspartate aminotransferase >70 U/L), and haemolysis characterised by serum lactic dehydrogenase level >600 U/L [17]. Preterm birth was defined as delivery before 37 completed weeks of gestation.

#### 2.3.2. Perinatal Outcome

Only major congenital malformations, defined as abnormalities that were fatal, required major surgery or resulted in severe organ malfunction or cosmetic defects were recorded and classified as related to the cardiovascular, central nervous, urogenital system, or other systems. Perinatal mortality was defined as fetal loss after 22 weeks of gestation or neonatal loss during the first 28 days after delivery. Large for gestational age (LGA) was defined as a birth weight above the 90th percentile corrected for gestational age, sex, and parity [18]. 

### 2.4. Statistical Analysis

Continuous parameters were expressed as mean ± SD when normally distributed and as median (*Q*1–*Q*3) in case of a skewed distribution. Categorical results were expressed as percentages. The appropriate (non)parametric tests (i.e., independent *t*-test when normally distributed and Mann-Whitney *U*-test in case of a skewed distribution) were used to compare differences between groups for continuous data and the chi-square or Fisher's exact test for categorical variables. A *P* value <0.05 was considered as statistically significant. All statistical analyses were performed using PASW for windows version 18.0 (SPSS, Inc., Chicago, IL, USA).

## 3. Results

A total of 287 singleton pregnancies in women with pregestational type 2 diabetes were referred to our centers. 15 pregnancies ended before 20 weeks of gestation, leaving 272 ongoing pregnancies and deliveries (11 in Amersfoort, 28 in Groningen, 72 in Utrecht, 65 in Amsterdam, 39 in Rotterdam, 10 in Heerlen, and 47 in the Hague). Data from 36 women from one of the centres have been published before [19].

### 3.1. Maternal Characteristics

Baseline data of the women are shown in Table 1. 80 pregnancies (29.4%) were from women of native Dutch origin and 192 pregnancies were from women of nonnative Dutch origin (70.6%). The specific origin of nonnative women with pregestational type 2 diabetes was North African in 73 (26.8%), Hindu in 59 (21.7%), Afro-Caribbean in 43 (15.8%), Asian in ten (3.7%) and, other nonnative in seven (2.6%) women. The mean age at conception was 33.0 ± 5.6 (range 18–48 years) and comparable between native and nonnative Dutch women (32.9 ± 4.8 versus 33.1 ± 5.9; *P* = 0.829). The median gestational age (GA) at first booking was significantly higher in the nonnative group compared to the native group (11.5 (8.0–19.0) versus 8.0 (6.0–10.3) weeks; *P* < 0.001). The clinical duration of diabetes was generally short. There was a significant difference in the use of medication between the two groups: about half of the native women were on insulin, whereas about three-quarters of the nonnative women were on oral glucose-lowering drugs or diet only at presentation (*P* < 0.001). The glycemic control was less favourable in nonnative women as compared to native women before and during pregnancy (*P* values 0.04 and <0.001, resp.), although in itself quite reasonable.

### 3.2. Maternal and Perinatal Outcome

Pregnancy outcome of the total study population and between native and nonnative Dutch women is shown in Table 2. In the total study population, median GA at delivery was 38 weeks; no data of delivery mode was available in one woman because she was referred to another hospital while in labour. Delivery started spontaneously in 75 (27.7%) women, labour was induced in 131 (48.3%), women and a primary Caesarean section (CS) was performed in 65 (24.0%) women. 49 of the 206 women (23.7%) who were planned to deliver vaginally had a secondary CS. The total CS rate was 42.1%. The incidence of CS was significantly higher in native Dutch women as compared to nonnative Dutch women. This difference was caused by a higher incidence of primary CS in native Dutch women with no difference in the incidence of secondary CS between the two groups. The other maternal outcome variables were not different between native and nonnative Dutch women.

*Perinatal Mortality*. 13 perinatal deaths (4.8%) occurred in the total study group, five fetal and eight neonatal deaths (Table 3). Four (31%) deaths were associated with congenital malformations; four (31%) infants died before or at a GA of 25 weeks and the remaining five (38%) deaths were due to infection (*n* = 1), premature rupture of the membranes (*n* = 2), tension pneumothorax (*n* = 1), or remained unexplained (*n* = 1). No differences in perinatal mortality reflecting maternal ethnicity were found.

*Congenital Malformations*. Major congenital malformations occurred in 17 (6.3%) infants of the total study population; the incidence in native and nonnative Dutch women was similar. Major congenital malformations involved the cardiovascular system (*n* = 7), the central nervous system (*n* = 4), urogenital system anomalies (*n* = 2), or other structures (*n* = 4). Three of these women were on oral glucose-lowering drugs during the first trimester.

*Large for Gestational Age*. 85 (31.3%) infants were large for gestational age in the total study population, with no differences between native and nonnative Dutch women.

## 4. Discussion

In contrast to our hypothesis, we showed no differences in pregnancy outcome between native and nonnative Dutch women with pregestational type 2 diabetes, with the exception of a higher CS rate in native Dutch women. However, a high incidence of adverse pregnancy outcomes in the complete group of women with pregestational type 2 diabetes living in the Netherlands was found.

Pregnancy outcome of women with pregestational type 2 diabetes has been studied before and our results are generally in accordance with those reports [5, 7–9]. According to the results of a study on ethnic differences in perinatal mortality in the Netherlands [13], we expected that outcome in nonnative women with type 2 diabetes would be poorer as compared to native Dutch women, for example, linked to cultural or knowledge barriers in some ethnic minorities affecting access to and effectiveness of care. However, we did not find significant differences in pregnancy outcome (except for the incidence of CS). These data are in accordance with studies from New Zealand and Europe [14, 15, 20, 21]. In the previous European studies, native Caucasians were compared with a single other group. In the study of Hughes et al. from New Zealand, three main ethnicities (i.e., Polynesian, Asian, and European women) were compared. There are some differences between these studies and ours. Firstly, we included all ethnicities, although the limited group size forced us to treat them statistically as one group. Secondly, none of these studies were truly nationwide, while the hospitals participating in our study were spread throughout the Netherlands. From these studies and from our data, it can be concluded that in a setting of easy access to and compliance with the local health care system, outcome in nonnative women with pregestational type 2 diabetes can be similar to that in native women with pregestational type 2 diabetes. This easy access to care might be positively affected by the fact that medical care, for example, in the Netherlands is fully reimbursed with insurance coverage for basically all inhabitants, resulting in an absence of any financial barriers to receive medical care. Apparently, potential negative effects of slightly poorer glycemic control and later presentation are offset by the care system. 

The only parameter which differed between native and nonnative Dutch women with pregestational type 2 diabetes was the higher incidence of a (primary) CS in the native group. This difference might partly be due to a higher incidence of maternal overweight, which according to US papers is related to the CS incidence [22, 23]. It may also be that women of native Dutch origin more often opted and negotiated for a primary CS.

The proportion native/nonnative Dutch (i.e., 29.4% versus 70.6%) was not representative for the normal Dutch population [24]. Firstly, this can be explained by a higher incidence of type 2 diabetes among specific non-European subpopulations, such as African, Asian, or Middle-Eastern populations [11]. Secondly, the hospitals that cooperated to this study are mainly located in the urban areas of the Netherlands, where the majority of nonnative Dutch people are living [25].

Nevertheless, the pregnancy outcome of all studied women still seems worse than the outcome of the general population. According to our study, ethnicity appears not to be involved in the development of this adverse outcome. Possibly, other factors play a pathophysiological role in this process and offer opportunities to improve this adverse outcome. Firstly, general awareness of the necessity of pregnancy planning in women with type 2 diabetes may be less than optimal in The Netherlands, since these women are generally seen in long-term community care and only referred to secondary care when already pregnant. Unfortunately, we were unable to report about the preconceptional care of the studied women, due to the retrospective nature of our study. Therefore, a prospective study about the effects of preconceptional care and pregnancy planning on pregnancy outcome in women with type 2 diabetes is needed. Secondly, postprandial spikes of the blood glucose levels contribute to a high-normal or a minimally elevated, but acceptable (42–53 mmol/mol), HbA1c level [26]. Nowadays, there are novel technical options to improve the glycemic control, like continuous glucose monitoring and insulin pumps [1, 27]. Thirdly, several studies indicate that a low socioeconomic status (SES) is associated with adverse pregnancy outcomes [13, 28, 29]. Since the incidence of type 2 diabetes is the highest in populations with a low SES [30], an etiological role of this “risk factor” may be expected in the development of complications during pregnancy among women with pregestational type 2 diabetes. Unfortunately, SES was not sufficiently recorded in our retrospective study and SES should, therefore, be analyzed in future prospective studies.

Limitations of our study are the retrospective nature, the limited size of the total study population and of the different ethnic groups, and the possibility of selection bias. Only 272 pregnancies were studied from seven large hospitals covering a representative area in The Netherlands, but possibly not fully representative, since four centres are tertiary care centres and three are larger secondary care centres. This might have led to a selection bias, since patients with a poor obstetrical history are referred to tertiary centres. 

In conclusion, this study has demonstrated that ethnicity seems not to be a major issue in the cause of pregnancy complications in women with pregestational type 2 diabetes with easy access to medical care. The results are still not approaching the pregnancy outcome of healthy, nondiabetic women. More prospective research is needed to validate these results and to pay more attention to possible causative factors like preconceptional care, avoiding postprandial spikes of the blood glucose and socio-economic status, in the development of adverse pregnancy outcome in women with pregestational type 2 diabetes.

## Figures and Tables

**Table 1:** General characteristics of the study population. *P* values were calculated between the two ethnic groups.

Maternal characteristics	Native Dutch, *n* = 80	%	Nonnative Dutch, *n* = 192	%	*P*
Mean (SD) age in yrs	32.9 ± 4.8		33.1 ± 5.9		0.829
Mean (SD) body mass index in kg/m^2^	32.1 ± 6.5		30.4 ± 5.6		0.096
Median (*Q*1–*Q*3) GA at 1st booking in weeks	8.0 (6.0–10.3)		11.5 (8.0–19.0)		<0.001
Alcohol use	0	0	0	0	n/a
Smoking habits	11	13.8	11	5.7	0.06
Nulliparous	22	27.5	34	17.7	0.079
Median (*Q*1–*Q*3) duration of diabetes (yrs)	2.0 (1.0–5.8)		1.0 (0.0–3.0)		0.004
Chronic complications					
Retinopathy	3	3.8	7	3.6	0.657
Neuropathy	1	1.3	2	1.0	0.65
Nephropathy	2	2.5	4	2.1	0.569
Cardiovascular	2	2.5	2	1.0	0.338
Preconceptional treatment of diabetes					<0.001
No	22	27.5	80	41.7	
Oral	9	11.3	64	30.3	
IIT	36	45.0	36	18.8	
CSII	6	7.5	1	0.5	
Unknown	7	8.8	11	5.7	
Glycemic control					
Median (*Q*1–*Q*3) HbA1c preconceptional	48 (39–59) (*n* = 25)*		52 (45–68) (*n* = 46)*		0.04
Median (*Q*1–*Q*3) HbA1c first trimester	45 (36–57) (*n* = 45)*		53 (43–66) (*n* = 107)*		0.001
Median (*Q*1–*Q*3) HbA1c second trimester	37 (34–43) (*n* = 47)*		45 (40–53) (*n* = 118)*		<0.001
Median (*Q*1–*Q*3) HbA1c third trimester	40 (34–44) (*n* = 49)*		42 (40–51) (*n* = 143)*		<0.001
Median (*Q*1–*Q*3) HbA1c during pregnancy	41 (36–48) (*n* = 49)*		48 (41–56) (*n* = 143)*		<0.001
Mean (SD) birth weight in grams	3395 ± 698		3376 ± 822		0.857

*Number of available HbA1c samples.

**Table 2:** Maternal and perinatal outcome of the total study population and between native and nonnative Dutch women with pregestational type 2 diabetes.

Complications	DM2 (*n* = 272)	MV	%	Native Dutch (*n* = 80)	MV	%	Nonnative Dutch (*n* = 192)	MV	%	*P* value
Maternal										
Preeclampsia/HELLP	29	17	11.4	11	1	13.9	18	6	9.7	0.162
Prematurity	52	0	19.1	16	0	20	36	0	18.8	0.811
Caesarean section	114	1	42.1	44	0	55	70	1	36.6	0.005
Primary	65	1	24.1	29	0	36.3	36	1	18.8	0.007
Secondary	49	1	18.1	15	0	18.8	34	1	17.8	0.797
Perinatal										
Perinatal mortality	13	0	4.8	3	0	3.8	10	0	5.2	0.607
Congenital malformations	17	3	6.3	7	1	8.8	10	2	5.3	0.269
Macrosomia	85	6	32.0	24	2	30.8	61	4	32.4	0.789

MV: missing values due to inappropriate data of follow-up.

**Table 3:** Characteristics of the 13 perinatal deaths in pregnancies of women with type 2 diabetes.

Case	Maternal origin	First trimester HbA1c (mmol/mol)	Mean HbA1c during pregnancy (mmol/mol)	Congenital malformations	Obstetric complications	Gestational age at delivery	Size at birth	Cause of death
1*	Nonnative	65	57	—	Cervical insufficiency	23	—	Prematurity
2*	Nonnative	54	56	—	—	25	AGA	Parvo infection
3*	Nonnative	—	45	—	—	24	SGA	Unexplained
4*	Nonnative	—	78	—	—	36	AGA	Unexplained
5*	Nonnative	80	60	Hypoplastic heart	—	35	LGA	Congenital malformation
6^†^	Native	—	46	Ventriculomegaly/ cerebral anomalies	Pre-eclampsia	37	AGA	Congenital malformation
7^†^	Native	—	49	Truncus Arteriosus type 2	—	38	AGA	Congenital malformation
8^†^	Nonnative	—	—	Cardiac malformation		37	SGA	Congenital malformation
9^†^	Native	51	46	—	Pre-eclampsia	30	AGA	Infection
10^†^	Nonnative	—	76	—	PROM	26	AGA	Prematurity
11^†^	Nonnative	—	—	—	Chorioamnionitis	22	SGA	Prematurity
12^†^	Nonnative	—	32	—	PROM	33	AGA	PROM
13^†^	Nonnative	69	64	—	Vacuumextraction	36	LGA	Tension pneumothorax

*Stillbirth, ^†^neonatal death, PROM: premature rupture of membranes, SGA: small for gestational age (<10th percentile), AGA: appropriate for gestational age, LGA: large for gestational age (>90th percentile).
